# Remdesivir increases mtDNA copy number causing mild alterations to oxidative phosphorylation

**DOI:** 10.1038/s41598-023-42704-y

**Published:** 2023-09-15

**Authors:** Nicole DeFoor, Swagatika Paul, Shuang Li, Erwin K. Gudenschwager Basso, Valentina Stevenson, Jack L. Browning, Anna K. Prater, Samantha Brindley, Ge Tao, Alicia M. Pickrell

**Affiliations:** 1 School of Neuroscience, Virginia Tech, Life Science I Room 217, 970 Washington Street SW, Blacksburg, VA 24061 USA; 2 Graduate Program in Biomedical and Veterinary Sciences, Virginia-Maryland College of Veterinary Medicine, Blacksburg, VA 24061 USA; 3 Department of Regenerative Medicine and Cell Biology, Medical University of South Carolina, Charleston, SC 29425 USA; 4 Department of Biomedical Sciences and Pathobiology, Virginia-Maryland College of Veterinary Medicine, Blacksburg, VA 24061 USA; 5 Virginia Tech Animal Laboratory Services, Virginia-Maryland College of Veterinary Medicine, Blacksburg, VA 24061 USA

**Keywords:** Drug safety, Mutation, Molecular medicine, Sequencing, Mitochondria

## Abstract

SARS-CoV-2 causes the severe respiratory disease COVID-19. Remdesivir (RDV) was the first fast-tracked FDA approved treatment drug for COVID-19. RDV acts as an antiviral ribonucleoside (adenosine) analogue that becomes active once it accumulates intracellularly. It then diffuses into the host cell and terminates viral RNA transcription. Previous studies have shown that certain nucleoside analogues unintentionally inhibit mitochondrial RNA or DNA polymerases or cause mutational changes to mitochondrial DNA (mtDNA). These past findings on the mitochondrial toxicity of ribonucleoside analogues motivated us to investigate what effects RDV may have on mitochondrial function. Using in vitro and in vivo rodent models treated with RDV, we observed increases in mtDNA copy number in Mv1Lu cells (35.26% increase ± 11.33%) and liver (100.27% increase ± 32.73%) upon treatment. However, these increases only resulted in mild changes to mitochondrial function. Surprisingly, skeletal muscle and heart were extremely resistant to RDV treatment, tissues that have preferentially been affected by other nucleoside analogues. Although our data suggest that RDV does not greatly impact mitochondrial function, these data are insightful for the treatment of RDV for individuals with mitochondrial disease.

## Introduction

As of December 2022, over half a billion people contracted severe acute respiratory syndrome coronavirus 2 (SARS-CoV-2), which causes the upper respiratory disease COVID-19 resulting in approximately, but most likely underestimated, 6.5 million deaths worldwide^1^. Considering the number of infections allowing for the mutagenesis of SARS-CoV-2, treatments for severe disease have been limited^2,3^. Currently, the United States Food and Drug Administration (FDA) has fully approved three medications for severe disease, Veklury™ (remdesivir) and two immunosuppressant therapies, Actemra™ (tocilizumab) and Olumiant™ (baricitinib). The first treatment remdesivir (RDV), a ribonucleoside analogue (adenosine), was first made available under Emergency Use Authorization, May of 2020^4,5^. The FDA directive allowed prescribing physicians to weigh the risks and benefits of this therapeutic treatment for use before its final approval in October 2020. Once approved, physicians in the US were using this ribonucleoside to reduce hospitalization times in efforts to avoid overpopulation and strain on the healthcare system^6^.

RDV was initially in development for the treatment of Ebola virus, but was also shown to be efficacious against SARS-CoV and Middle East respiratory syndrome coronavirus (MERS-CoV), preventing replication and ameliorating disease^7^. Remdesivir is nonspecifically, intracellularly catalyzed into its active form, remdesivir-triphosphate, an analogue of adenosine tri-phosphate^8^. Remdesivir then works by delayed chain termination inhibiting viral RNA synthesis^9,10^.

Mitochondria are of endosymbiotic origin^11^, which uniquely places this organelle at risk for off-target side effects affecting mitochondrial function during both antibiotic and antiviral treatments. Mitochondria contain multiple copies of their own ~ 16.5kB double stranded DNA inside each mitochondrion within the cell. Mitochondrial DNA (mtDNA) encodes for 13 proteins required for oxidative phosphorylation (OXPHOS) and ATP generation for the cell alongwith its own ribosomal and mRNA machinery to transcribe and translate these polypeptides^12^. Mutations, deletions, or mtDNA copy number depletion causes defects in OXPHOS leading to energy failure and tissue dysfunction^13,14^. Antiviral analogs have previously been found to disrupt the function of the mitochondrial DNA-directed RNA polymerase (POLMRT) (transcription, mtDNA replication), the mtDNA polymerase gamma (replication), and thymidine kinase 2 (mtDNA nucleotide recycling)^15^.

Previously, antiviral drugs, specifically nucleoside analogues, demonstrated off-target effects on mitochondrial function. During the US HIV/AIDS epidemic in the 1980 and 1990’s, a thymidine analogue designed to treat HIV patients, zidovudine (AZT), was identified, moved into Phase I clinical trials, and was approved by the FDA in a 3-year span from 1984 to 1987^16,17^. AZT was approved in record time with only one 19-week human clinical trial^18^. Undeniably, AZT and newer generation antivirals that came after the development of AZT led the way to better treatment and management strategies for HIV/AIDS, turning a deadly disease into a chronic yet manageable disease. However, research studies years later demonstrated the negative effects AZT exposure had on mitochondrial function^19,20^ and was verified to contribute to premature tissue aging and myopathies in AZT-treated patients^21,22^. These effects were also seen with short-term usage impairing the respiratory chain^23–25^. AZT has also been shown to cross the placenta in nonhuman primates^26^. Findings in both nonhuman primates and human infants have found AZT may have negative effects on mitochondria and mtDNA causing additional concern for their usage^27,28^. Due to these past findings, we decided to test whether remdesivir inadvertently caused similar off-target effects on mitochondrial function. Here, we report that mild changes to mtDNA occur in response to RDV treatment, but mitochondrial function is largely unperturbed in acute regimens used in this study.

## Results

### Mv1Lu cells increase mtDNA copy number in response to remdesivir with minimal changes to oxidative phosphorylation

**Figure 1:**
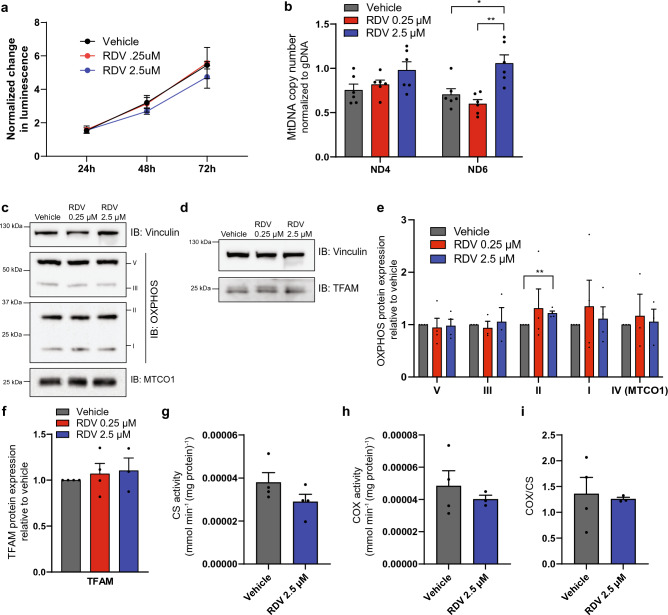
Mv1Lu cells increase mtDNA copy number in response to remdesivir with minimal changes to oxidative phosphorylation. (**a**) Normalized luminescence measurements for viability using CellTiter-Glo for Mv1Lu cells exposed to RDV. n = 4 independent experiments. (**b**) MtDNA copy number quantification for Mv1Lu cells exposed to RDV using two primer sets against the mtDNA normalized to guide DNA (gDNA) levels. (**c**) Representative western blots for subunits for oxidative phosphorylation complexes and MTCO1. Vinculin is used as a loading control. Full-length membrane images can be found in Figure S3a-d. (**d**) Representative western blots for TFAM. Vinculin is used as a loading control. Full-length membrane images can be found in Figure S3e,f. (**e**) Quantification of western blots in (**c**) for subunits for oxidative phosphorylation complexes normalized to vinculin. Protein changes in RDV treated cells are normalized to vehicle. (**f**) Quantification of western blots for TFAM in (**d**) normalized to vinculin. Protein changes in RDV treated cells are normalized to vehicle. (**g,h**) Spectrophotometer assays for (**g**) citrate synthase activity and (**h**) complex IV activity normalized to protein. (**i**) Ratio of complex IV activity to citrate synthase activity. Error bars ± SEM. * = *p* < 0.05; ** = *p* < 0.01. One dot equals an experimental replicate.

Mv1Lu were chosen to first test whether RDV caused off-target effects in vitro. SARS-CoV-2 uses angiotensin-converting enzyme 2 (ACE2) as an entry receptor into the host cell^29^, which is highly expressed in lung and airway epithelia^30^. These cells are of epithelial origin and have been shown to support the replication of coronaviruses^31^. Mv1Lu cells were treated with either a high (2.5 μM) or low (0.25 μM) dose of remdesivir for 72 h. 0.25 μM or 2.5 μM were concentrations that previously showed efficiency in cell culture when clearing SARS-CoV-2 virus^32,33^. While the viability of these cells was unaffected at either dose (Fig. 1a), a significant, slight increase in mtDNA copy number occurred with a high dose of remdesivir with primers targeting the *ND6* region (Fig. 1b). However, this result was not recapitulated using another mtDNA primer set against the mtDNA region of the gene *ND4* (Fig. 1b). We next set out to test whether mitochondrial function was perturbed by RDV. Oxidative phosphorylation (OXPHOS) protein expression showed mostly no changes except an increase in the nuclear-encoded succinate dehydrogenase [ubiquinone] iron-sulfur (SDHB) subunit of complex II for cells treated with 2.5 μM of RDV (Fig. 1c,e). Other subunits were unaffected, which were also nuclear-encoded, for complexes I (NDUFB8), III (UQCRC2), and V (ATP5A) (Fig. 1c,e). This was also true for the mtDNA-encoded cytochrome *c* oxidase (COX) 1 subunit (Fig. 1c,e), indicating that changes to mtDNA copy number did not result in a biological effect. Mitochondrial transcription factor A (TFAM), which is responsible for mtDNA replication and transcription, was also unaffected by RDV treatment (Fig. 1d,f). Considering that complex IV activity is sensitive to mtDNA alterations^34,35^, we then tested the activity of citrate synthase (CS) and cytochrome *c* oxidase (complex IV) finding no effect of remdesivir treatment (Fig. 1g–i).

### MtDNA copy number is increased in the liver of mice treated with remdesivir, but doesn’t impact liver function

**Figure 2:**
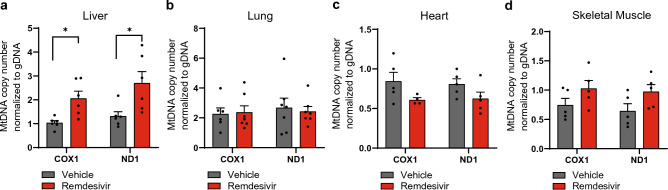
MtDNA copy number is increased in the liver of mice treated with remdesivir. (**a-d**) MtDNA copy number quantification for CD-1 male mice treated with remdesivir for 10 days, 30 days post-treatment, using two primer sets against the mtDNA normalized to gDNA levels. Tissues isolated for analysis were (**a**) liver (**b**) lung (**c**) heart and (**d**) skeletal muscle. Error bars ± SEM. * = *p* < 0.05. One dot equals an individual animal.

It was possible that slight changes in vitro could be magnified in vivo. Also, previous studies demonstrate that post-mitotic tissues like heart and skeletal muscle accumulate more mtDNA mutations after nucleoside analogue treatment perhaps because these cells cannot turn over^21,36,37^. We treated 2-month-old adult CD-1 male mice for 10 days with remdesivir, choosing a duration more likely to mimic the treatment provided to human patients with COVID-19^38^. We chose male animals because more men were enrolled in the initial clinical trials for RDV^39^, and more men were hospitalized for COVID-19 (60.3–39.7%) than women^40^. The initial reports on RDV also indicated a 10-day treatment period for COVID-19 provided benefit to patients over placebo^6^, so we also chose a 10-day treatment regimen using efficacious doses previously reported in mice^41^. We then measured mtDNA copy number levels using two primer sets targeting the regions encoding the genes for *ND1* and *COX1* in liver, lung, heart, and skeletal muscle. We found a significant increase in mtDNA copy number 30 days post-treatment in liver using both primer sets (Fig. 2a). However, there were no detectable changes to mtDNA copy number for lung, heart, and skeletal muscle (Fig. 2b–d).

**Figure 3:**
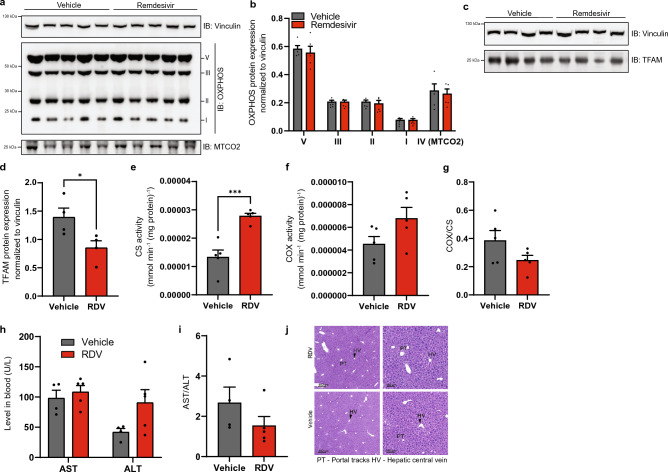
Liver shows mild changes to mitochondrial function when treated with remdesivir. (**a**) Representative western blots for subunits for oxidative phosphorylation complexes in liver. Vinculin is used as a loading control. Full-length membrane images can be found in Figure S4a-c. (**b**) Quantification of liver tissue western blots in (**a**) for subunits for oxidative phosphorylation complexes normalized to vinculin. (**c**) Representative western blot for TFAM in liver. Vinculin is used as a loading control. Full-length membrane images can be found in Figure S4d,e. (**d**) Quantification of liver western blots for TFAM in (**c**) normalized to vinculin. (**e–f**) Spectrophotometer assays for (**e**) citrate synthase activity and (**f**) complex IV activity normalized to protein. (**g**) Ratio of complex IV activity to citrate synthase activity. (**h**) Blood serum testing for AST and ALT enzyme levels. (**i**) De Ritis ratio of AST/ALT levels. (**j**) Representative hematoxylin and eosin staining images of liver collected 30 days post-treatment. Scale bar = 400 μm for left images. Scale bar = 100 μm for right images. RDV n = 4, vehicle n = 5. Error bars ± SEM. * = *p* < 0.05; *** = *p* < 0.001. One dot equals an individual animal.

With this change in mtDNA copy number, we next decided to profile whether mitochondrial function was altered after treatment in the liver. Surprisingly, western blots probing for oxidative phosphorylation subunits showed no change with RDV treatment compared to controls (Fig. 3a,b), but TFAM protein expression in liver was decreased (Fig. 3c,d). Steady state protein levels can be relatively stable for nuclear and mitochondrial DNA subunits unless a severe mitochondrial defect is detected, so we performed spectrophotometer assays probing CS, an indicator of mitochondrial mass not reliant on OXPHOS, and complex IV activity. CS activity (Fig. 3e) shows a significant increase in activity with RDV treated liver samples, which elevated mtDNA copy number could indicate increased mitochondrial biogenesis to compensate for mitochondrial defects. However, both complex IV activity normalized to protein and the COX/CS ratio remained unaffected by RDV (Fig. 3f–g).

We next profiled liver function to test whether RDV was toxic to the liver, since some changes to mitochondrial function were found. Heightened levels of liver enzymes aspartate transaminase (AST) and alanine transaminase (ALT) in blood serum, as well as the De Ritis ratio are indicative of liver damage. No significant changes in AST, ALT, or the De Ritis ratio were detected in the blood of RDV treated mice (Fig. 3h–i). In line with these findings, no changes in histology of the liver sections were observed (Fig. 3j). Small areas of extramedullary hematopoiesis were seen multifocally within sinusoids of liver section on both groups, which is considered a common incidental finding.

**Figure 4:**
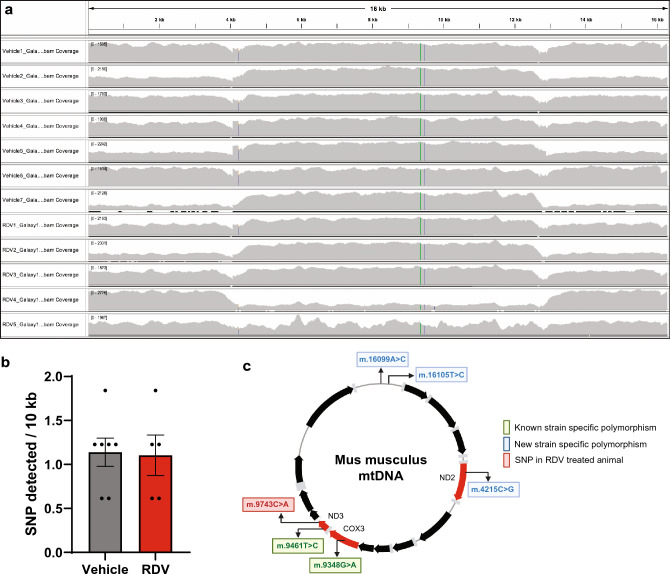
Next-generation sequencing finds no change in mutational load to liver after remdesivir treatment. (**a**) IGV snapshot of sequencing depth and coverage maps. (**b**) Quantification of single nucleotide polymorphisms (SNPs) detected per 10 kilo base-pairs per sample. One dot equals an individual animal. (**c**) Cartoon depiction mapping polymorphisms found in NGS data on the mouse mitochondrial genome. Biorender.com was used in the generation of this panel.

Considering that CS activity was increased as well as mtDNA copy number, we performed next-generation sequencing (NGS) to test whether mutational load was affected after RDV treatment in liver. Mutations in the control region that may affect TFAM binding may explain why TFAM and mtDNA levels did not positively correlate with each other. Sequencing coverage for the mtDNA spanned the whole mitochondrial genome for both groups with a uniform depth of coverage (Fig. 4a). Although the mutational load did not differ between the groups **(**Fig. 4b), we identified three novel polymorphisms that differed from CD-1 and mouse reference genome publicly available on NCBI. In just one RDV treated mouse, a point mutation, (m.9743C > A) in the *ND3* gene region was found that was not present in any of the vehicle samples (Fig. 4c). Overall, RDV did not have the same mutagenic effect as other antiviral ribonucleosides 30 days post treatment.

### Lung is unaffected by remdesivir treatment

**Figure 5:**
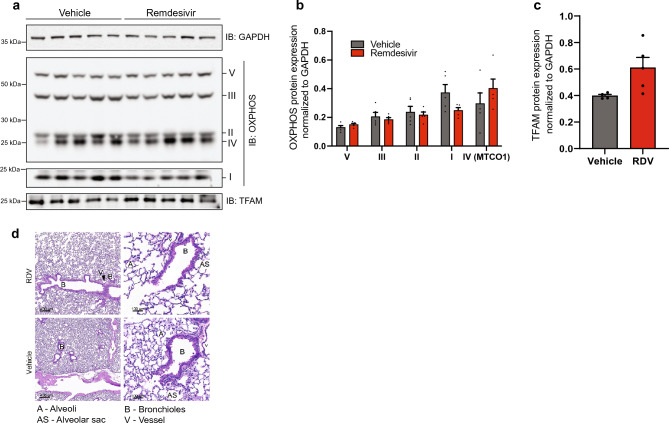
Lung is unaffected by remdesivir treatment. (**a**) Representative western blots for subunits for oxidative phosphorylation complexes in lung. GAPDH is used as a loading control. Full-length membrane images can be found in Figure S5a-d. (**b**) Quantification of lung western blots in (**a**) for subunits for oxidative phosphorylation complexes normalized to GAPDH. (**c**) Representative western blot for TFAM in lung. GAPDH is used as a loading control. (**d**) Quantification of lung western blots in (**c**) for TFAM normalized to GAPDH. (**e**) Representative hematoxylin and eosin staining images of lung collected 30 days post-treatment. Scale bar = 400 μm for left images. Scale bar = 100 μm for right images. RDV n = 2, vehicle n = 4. Error bars ± SEM. n = 5/group. One dot equals an individual animal.

While no changes were detected in mtDNA copy number in lung (Fig. 2b), we tested whether lung tissue appeared affected by RDV treatment. All of the subunits of oxidative phosphorylation probed for on western blot (Fig. 5a,b) and TFAM expression (Fig. 5a,c) remained unchanged with RDV treatment compared to controls. Pathology of hematoxylin and eosin stained lung tissue sections showed no histological differences as well (Fig. 5d).

### Cardiac function is unaffected by remdesivir

Previous studies have found the nucleoside analogue, AZT, to have a detrimental effect on cardiac and skeletal muscle, but we did not detect any changes to mtDNA copy number in these tissues (Fig. 2c,d). We performed western blotting to probe for subunits of OXPHOS finding no significant changes in either heart (Figure S1a-b) or skeletal muscle (Figure S1c-d) in our treatment groups.

**Figure 6:**
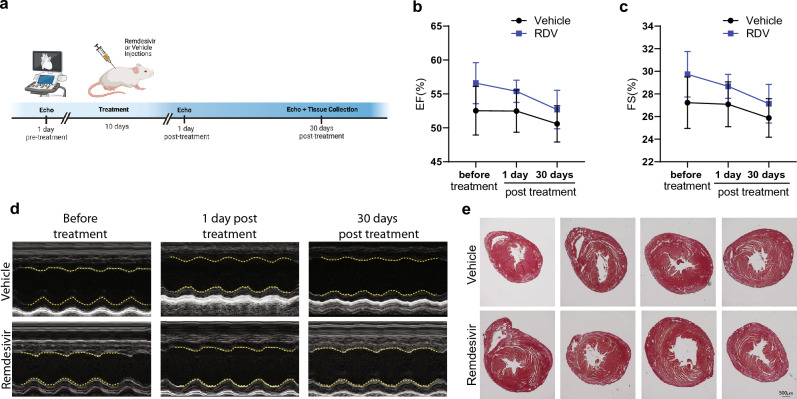
Remdesivir does not cause functional deficits to cardiac function. (**a**) Timeline diagram showing drug treatment experimental paradigm for echocardiograms performed prior and post-treatment. Biorender.com was used in the generation of this panel. (**b**,**c**) Echocardiography showing (**b**) ejection fraction and (**c**) fractional shortening. (**d**) Representative images of echocardiography of each group. Left ventricle inner wall is depicted by yellow dashed lines. (**e**) Representative trichrome staining images of hearts 30 days post-treatment after echocardiography. Scale bar = 500 μm. Error bars ± SD. n = 10/group.

To ensure no change in cardiac function was detected, echocardiograms were measured at three timepoints: 1 day pre-treatment, 1 day post-treatment, and 30 days post-treatment (Fig. 6a). Ejection fraction and fractional shortening did not differ between groups at all the time points tested (Fig. 6b–d). Trichrome staining for histology also shows no cardiac abnormalities in RDV treated mice (Fig. 6e).

## Discussion

Remdesivir was originally developed for hepatitis C, repurposed for the Ebola and Marburg viruses, and now is utilized for SARS-CoV-2^5^. Antiviral nucleosides will continue to be recycled and reused against emerging viral threats until researchers generate more targeted combinatory therapies to a specific viral strain. Even then, the fast adaptation and mutational ability of viruses such as SARS-CoV-2^42^ will still necessitate the usage of these broad antiviral treatment strategies. Our data suggests that remdesivir does have the ability to impact mitochondrial DNA and affect mitochondrial function, albeit not to the extent that it greatly impacts tissue physiology under these experimental conditions.

Previous in vitro data suggests that mitochondrial RNA polymerase does have the ability to incorporate remdesivir; however, its selectivity for ATP against remdesivir-TP is around 500-fold^43^. Previous studies evaluated whether mitochondrial toxicity occurs in cell culture. Studies using immortalized cell lines found mitochondrial alterations and mtDNA copy number depletion but only at high micromolar concentrations that were cytotoxic^44,45^. However, hiPSC-CMs differentiated into iCMs displayed fragmented mitochondria, depletion of mtDNA encoded RNAs, and defects in respiration and ATP levels when exposed to RDV at 2.5 uM concentrations^41^. The pharmacokinetics of RDV can affect all tissues but brain showing the highest tissue distribution in the liver and kidneys^46^, but side effects reported for RDV such as hypotension and bradycardia have been reported in clinical trials^6,47^. Our data in vivo did not show an effect on the heart (Fig. 2c), but it is possible that time points past 30 days or a longer duration of RDV would have a different effect. Tissue distribution may also explain our findings in liver (Fig. 2a) and future studies should closely evaluate the effect RDV has on the kidneys. Our in vitro work in Mv1Lu cells used concentrations that did not affect cell viability (Fig. 1a) even at micromolar concentrations, and we found only significant effects on mtDNA copy number and SDHB protein levels but those did not significantly impact mitochondrial function. However, it is important to note, that repeated RDV 100 mg therapeutic doses in healthy human subjects do reach micromolar concentrations in the plasma^8^.

Previous work has already suggested or shown that SARS-CoV-2/coronaviruses localize to mitochondria in the host cell^48,49^. Other RNA viruses have been shown to affect mtDNA and mitochondrial RNA transcripts^50^. In the case of RNA viruses ZIKA and HSV-1, mitochondrial abnormalities have been found due to the viruses’ localization to the mitochondria^51,52^. This raises important issues not addressed in this study or previous studies: In the presence of SARS-CoV-2, will mitochondria be affected and will antivirals compound any dysfunction? It is also possible that with the viral load being reduced with antivirals, they might affect mitochondria to a lesser degree.

In vivo analysis of RDV treatment on mitochondrial function has yet to be extensively profiled. One study treated male, 30-month-old rats for 3 months finding no mtDNA copy number alterations or deletions in heart, kidney, or skeletal muscle^53^. Here we found mtDNA copy number was elevated in liver with concomitant increases in CS activity, but overall liver function appeared unaffected. Our results are largely in agreement; however, the duration between studies differs as well as the follow-up analysis on mitochondrial function and tissue function. We also chose male mice for our study as more men were being hospitalized with SARS-CoV-2 (60.3–39.7%) than women^40^. In addition, mitochondrial function is reported to be higher in multiple tissue and cell types in females as compared to males^54,55^, indicating that female physiology may be more resistant after exposure to nucleoside analogue treatments. One finding that was surprising is that TFAM levels did not positively correlate with mtDNA levels (Figs. 2a and 3c,d) considering TFAM is important for mtDNA replication and packaging^56^. NGS sequencing did not find any mutations in the TFAM binding region that could explain this result. We also cannot rule out that although the decrease was significant, it did not reach a threshold required to cause a biological effect. The duration of a more chronic treatment regime could be utilized if RDV is used for other viral infections, but a longer treatment duration was not considered for our study because it did not reflect treatment regimens for COVID-19^38^.

Preclinical evaluation is important. Another nucleoside analogue, fialuridine (FIAU), displayed promise in treating chronic hepatitis B infections^57,58^. However, during the Phase II clinical trial, the drug caused hepatic failure, lactic acidosis, and pancreatitis, which was fatal for five of the thirteen enrolled patients^59^. Studies later showed off-target effects included a decrease in mtDNA copy number^60^ and the enlargement of mitochondria with abnormal cristae^61^. In conclusion, our data suggests that caution may still be warranted for individuals with mitochondrial disease or mitochondrial defects when choosing RDV as a potential antiviral treatment.

## Materials and methods

### Cell culture

Mv1Lu cells (ATCC) were grown in high glucose (25 mM) DMEM media (Gibco) with 10% FBS (Gibco), 1% HEPES (Gibco), 1% sodium pyruvate (Sigma-Aldrich), 1% MEM Non-Essential Amino Acids (Gibco) and 1% L-Glutamine (Gibco). Cells were routinely tested for mycoplasma contamination by PCR (Southern Biotech).

### Chemicals

Experimental cells received a media change every 24 h with complete media with either a 2.5 μM or a 0.25 μM dose of remdesivir (Cayman Chemicals) in DMSO (Fisher). Vehicle treated cells received complete DMEM media change with DMSO.

### Animals

All mice were housed in pathogen-free facility on a 12-h light/dark cycle at Virginia Tech or Medical University of South Carolina and provided standard rodent diet and water ad libitum. 1.5-month-old ICR (CD-1®) outbred mice were purchased from Envigo. All experiments were conducted in accordance with the NIH Guide for the Care and Use of Laboratory Animals and ARRIVE guidelines, as well as under approval of the Virginia Tech Institutional Animal Care and Use Committee or Medical University of South Carolina Institutional Animal Care and Use Committee.

2-month-old male mice were injected for 10 consecutive days with either 12% sulfobutyl-b-cyclodextrin vehicle (Sigma-Aldrich) or 25 mg/kg dose of remdesivir (MedKoo) diluted in vehicle. Mice rested for 30 days with no treatment. Mice were deeply anesthetized with an intraperitoneal injection of ketamine (500 mg/kg) and xylazine (10 mg/kg) before sacrifice. Mice were perfused with ice cold 1 × PBS. Liver, lung, cardiac, and skeletal muscle (quadriceps femoris) tissue were flash frozen in liquid nitrogen and stored immediately at − 80 °C. Liver and lung were incubated in 10% formalin (VWR) in histology cassettes for paraffin blocks for hematoxylin and eosin staining performed by the Virginia-Maryland College of Veterinary Medicine Pathology core on 10 μM thick sections.

### Western blotting

Homogenized tissue or Mv1Lu cell pellets were resuspended in 1 × RIPA Lysis and Extraction Buffer (Thermo Scientific) with Pierce™ protease and phosphatase inhibitors. Samples incubated on ice for 20 min and then incubated at 4 °C end over end for 20 min. Samples were centrifuged at 16,000 × *g* for 15 min at 4 °C. Supernatant was collected and protein concentration was determined using the DC™ Protein Assay Kit II (Bio-Rad).

20–40 μg of protein in 2 × LDS-sample buffer (ThermoFisher) and 50 mM DTT (Sigma-Aldrich) were heated at 70 °C for 10 min. Samples were loaded onto NuPAGE™ 4 to 12% Bis–Tris gels (Invitrogen). Electrophoresis was run with 1 × MOPS-SDS Running Buffer (Bioworld) and transferred to a PVDF membrane (EMD Millipore) at 100 V for 1 h. Membrane was blocked with 5% milk for 1 h at RT. Primary antibodies were incubated overnight at 1:1000–1:5000 dilutions. Membranes were washed with 1 × TBST and incubated with secondary antibody conjugated to HRP for 1 h at RT. Membranes were imaged using the ChemiDoc (Bio-Rad) with Clarity™ Western ECL Substrate (Bio-Rad), ECL Select™ Western Blotting Detection Reagent (Cytiva), or SuperSignal™ West Femto Maximum Sensitivity Substrate (Thermo Scientific). Detected bands were quantified using ImageLab (Bio-Rad).

### Antibodies

The following antibodies were used for this study: anti-Vinculin (Invitrogen, # 700,062), OxPhos Human WB Antibody Cocktail (Invitrogen, # 45-8199), OxPhos Rodent WB Antibody Cocktail (Invitrogen, #45-8099), anti-TFAM (Sigma-Aldrich, #ABE483), anti-GAPDH (Sigma-Aldrich, #G9545), anti-COXII (Abcam, #ab198286), MTCO1 Monoclonal Antibody (Invitrogen, # 459,600), goat anti-mouse IgG (H + L) HRP conjugated (Jackson ImmunoResearch), and goat anti-rabbit IgG Antibody, (H + L) HRP conjugated (Jackson ImmunoResearch).

### DNA extraction

DNA extraction for Mv1Lu pellets were performed using the Quick-DNA™ Miniprep Kit according to the manufacturer’s instructions (Zymo). DNA extraction for tissue began with homogenization in 1 × PBS with Pierce™ protease and phosphatase inhibitors (Thermo Scientific). Samples were incubated overnight at 37 °C in RSB Buffer (10 mM Tris–HCl pH 7.4, 10 mM NaCl, 25 mM EDTA pH 8.0) in addition to 1 mg/mL Proteinase K (ApexBio), 1% SDS, and 0.2 mg/mL RNAseA (Fisher Scientific). DNA was extracted using phenol:chloroform and precipitated in 100% isopropanol (Fisher Scientific). 70% ethanol was added to pelleted DNA and spun down at 13,000 × *g* for 5 min. Pellets were dried at RT for 20 min. DNA was resuspended in nuclease-free water. DNA was then cleaned using the DNA Clean and Concentrator Kit (Zymo) according to the manufacturer’s instructions.

### qPCR for mtDNA copy number

10 ng of genomic DNA and 0.4 μM of each primer set was mixed in a 10 μL qPCR reaction that was run on the CFX96 System (Bio-Rad). Reactions were performed using PowerUp™ SYBR™ Green Master Mix (Applied Biosystems). The primers that were used are as follows: *ACTG1* (gDNA) (F: CGCAAGTACTCCGTGTGGAT, R: CAACTGCTACTCCGGGTTCG) *ND4* (mtDNA) (F: AGCCTTTACTCTATCTTTTATGGGA, R: ATAAGCCCAGTGCTGCTTCA) *ND6* (mtDNA) (F: CAATTCCACAGCCAATAGCCC, R: ACAACGGTGATTTTTCATGTCACT). *β-Actin* (F: GCGCAAGTACTCTGTGTGGA, R: CATCGTACTCCTGCTTGCTG), *COX1* (mtDNA) (F: AGGCTTCACCCTAGATGACACA, R: GTAGCGTCGTGGTATTCCTGAA) and *ND1* (mtDNA) (F: CAGCCTGACCCATAGCCATA, R: ATTCTCCTTCTGTCAGGTCGAA).

PCR was performed in technical triplicates. Data was collected from at least three independent experiments or the number of animals noted in the figure legend. Expression levels were normalized to genomic DNA and fold change was determined by comparative CT method^62^.

### Cytochrome c oxidase assay

Complex IV activity was measured as previously described^63^. A buffer comprised of 10 mM potassium phosphate pH 7.0, 1 mg/ml BSA (Gold Biotechnology), and 120 mM lauryl maltoside (Sigma-Aldrich) was added to tissue homogenates and cell pellets. 2 mM cytochrome *c* (Sigma-Aldrich) reduced with sodium dithionite (Fisher) was added to catalyze the reaction. Measurements were taken at 550 nm at 30 s intervals for 20 min at 37 °C. Potassium cyanide (240 μM) was used to inhibit the reaction to ensure slope was specific to COX. Results were normalized to protein concentration using the DC™ Protein Assay Kit II (Bio-Rad).

### Citrate synthase assays

Citrate synthase activity was measured in tissue and cell samples using the Citrate Synthase Assay Kit according to the manufacturer’s instructions (Abcam). Results were normalized to protein concentration using the DC™ Protein Assay Kit II (Bio-Rad).

### Cell viability

Approximately 300 to 600 Mv1Lu cells were plated (4 wells/treatment) in white-coated 96-well plates (Brand Tech Scientific) in growth media. Cell growth curve was obtained by CellTiter-Glo® Luminescent Cell Viability Assay (Promega) using a luminescence reader every 24 h. Mean cell number corresponding to the luminescence on each day was normalized to the first day in the graph.

### Liver enzymes

Blood from mice was collected via cardiac puncture. Mice were deeply anesthetized with an intraperitoneal injection of ketamine (500 mg/kg) and xylazine (10 mg/kg) prior to collection. 400 uL of blood from each mouse placed in a Microtainer Blood Collection Tube with Lithium Heparin (BD) was sent to Virginia-Maryland College of Veterinary Medicine for AST and ALT testing.

### Histological scoring

Liver and lung were collected after transcardial perfusion, fixed in 10% formalin (VWR) and embedded in paraffin blocks. Sections of 5 μm were stained with hematoxylin and eosin by the ViTALS group at Virginia-Maryland College of Veterinary Medicine. The control group contained 5 slides with 1 tissue section of liver, and 4 slides with 1–3 sections of lung. The treatment group contained 4 slides with 1 section of liver and 2 slides with 1 section of lung. Tissue sections were analyzed in a post-examination method of masking by an anatomic pathologist following adapted guidelines from the INHAND-recommended grading scheme^64^ for sections of the liver, and reported grading system that asses the qualitative presence of lesions based on distributions of the lung^65^. Briefly, tissue sections were screened for evidence of cellular degeneration, injury, cell death, or proliferative lesions. Findings from sections of the liver are categorized as within expected limits (no lesions), marginal (very small amount), slight (small amount), moderate (medium amount), marked (large amount), and severe (very large amount). Whereas findings from lung sections are categorized as 0% (none), < 25% (1), 26–50% (2), 51–75% (3), and > 75% (4), based on lung fields. Slides were digitally scanned using MoticEasy Scan (Motic) Infinity 60, and representative tissue sections were used for figure generation.

### Echocardiography

Echocardiography was performed using a Vevo 3100 ultrasound system (Fujifilm VisualSonics), equipped with a MS550S transducer, B-mode and M-mode datasets as previously described^66^.

### Trichrome staining

Mouse hearts were collected, fixed in 10% formalin (Leica Biosystems) overnight and embedded in paraffin. We prepared 7 μM sections and carried out trichrome staining as previously reported^67^.

### Next-generation sequencing

Mitochondrial DNA was purified from whole genome DNA from CD-1 liver tissue using KAPA HiFi HotStart ReadyMix (Roche) with 10 ng/μL DNA using two mitochondria primer sets that spanned the mtDNA genome: 4075F: AGCAGCAACAAAATACTTCGTCACAC, 12886R: GTGAGGGCGAGGTTCCGATTAC; 12728F: CTGTACCCACGCATTCTTCA, 4200R: GGATAGGCCTATTAATGTTATGT. PCR products were extracted using the GeneJET Gel Extraction Kit (Thermo Scientific).

Sequencing was performed at the Genomics Sequencing Center which is part of the Fralin Life Science Institute at Virginia Tech. DNA-seq libraries were constructed using KAPA HyperPrep Kit (Roche). For library preparation, input mtDNA was quantitated using a Qubit 3.0 (Thermo Fisher). Samples were then sheared using a Covaris M220 incident power of (W) 50, duty factor of 20%, cycles per burst of 200, and treatment time of 130 s in 50 μl. End repair and A tailing were performed on the roughly 100 ng of input DNA. Adapter ligation and barcoding were performed followed by a bead clean up and PCR (4 cycles). Agilent TapeStation was used to visualized the final libraries which were quantitated using Quant-iT dsDNA HS Kit (Invitrogen). Libraries were then normalized and pooled and sequenced on a MiSeq Nano 500 cycle, 250 paired end.

### Next-generation sequencing data analysis

All pipeline for NGS data analysis were performed using the usegalaxy.org public server^68^. Illumina universal adaptor sequences were removed from the fastq files using Cutadapt (Galaxy Version 4.0_galaxy1). Quality of the reads in all paired end fastq files were checked using FastQC (Galaxy Version 0.73 + galaxy0) before proceeding for read mapping. The reads were mapped using BWA-MEM2 (Galaxy Version 2.2.1 + galaxy0) to the reference mouse mitochondrial genome sequence (NC_005089.1). Depth and coverage quality for each sorted bam files were visualized using IGV_2.16.0 tool. FreeBayes Bayesian genetic variant detector (Galaxy Version 1.3.6 + galaxy0) with minimum depth of coverage of 10 was used for frequency-based pooled calling with filtering. Variant annotation with mouse genome build mm10 was done using SnpEff eff (Galaxy Version 4.3 + T.galxy2) tool.

### Statistical analysis

For comparisons between two groups, student’s t-test was used to determine statistical significance. Ordinary one-way ANOVA followed by Tukey’s multiple comparisons were used for three or more groups. All the graphs were plotted using Prism software. Differences in means were considered significant if *p* < 0.05 and designated as the following *p* < 0.05—*; *p* < 0.01—**; *p* < 0.001—***. The number of experimental replicates are included in the figure legends. One dot represents an individual animal or an experimental replicate.

### Supplementary Information

Supplementary Figures.

## Data Availability

The western blot data generated during this study are available at Mendeley Data https://doi.org/10.17632/phcxk2fw42.1. Next-generation sequencing data generated is available at NCBI BioSample database under SUB12974528, released upon publication. Any other raw datasets generated during this study are currently being used for future studies and to obtain grant funding, but data is available upon request.

## Abbreviations

ALT: Alanine transaminase
ACE2: Angiotensin-converting enzyme 2
AST: Aspartate transaminase
CS: Citrate synthase
COVID-19: Coronavirus disease 2019
COX: Cytochrome *c* oxidase
FIAU: Fialuridine
gDNA: Genomic DNA
MERS-CoV: Middle East respiratory syndrome coronavirus
mtDNA: Mitochondrial DNA
POLMRT: Mitochondrial DNA-directed RNA polymerase
TFAM: Mitochondrial transcription factor A
NGS: Next-generation sequencing
OXPHOS: Oxidative phosphorylation
RDV: Remdesivir
SARS-CoV-2: Severe acute respiratory syndrome coronavirus 2
SNP: Single nucleotide polymorphism
SDHB: Succinate dehydrogenase [ubiquinone] iron-sulfur subunit
FDA: United States Food and Drug Administration
AZT: Zidovudine
