# Supplementary material for: Remdesivir increases mtDNA copy number causing mild alterations to oxidative phosphorylation
Source: Sci Rep. 2023 Sep 15;13:15339. doi: 10.1038/s41598-023-42704-y (PMC10504289; doi:10.1038/s41598-023-42704-y)
Supplement: Supplementary file 1 — Supplementary Figures. [file 41598_2023_42704_MOESM1_ESM.pdf]

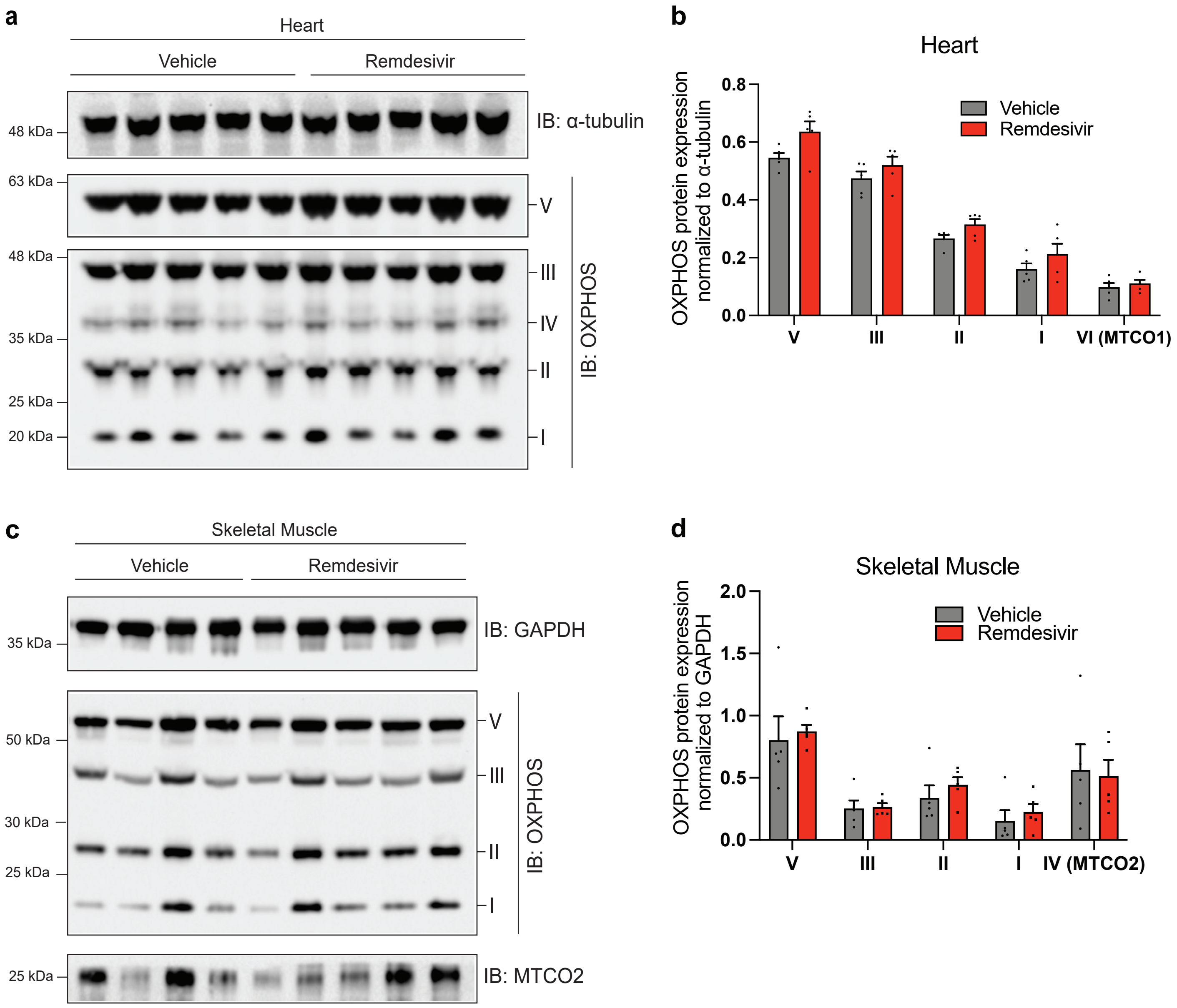

Supplementary Figure 1: Mitochondrial protein expression in heart and skeletal muscle is unaffected by remdesivir.

a) Representative western blot for subunits for oxidative phosphorylation complexes in heart. GAPDH is used as a loading control. Full-length membrane images can be found in Figure S6a,b. b) Quantification of heart western blots in (a) for subunits for oxidative phosphorylation complexes normalized to GAPDH. c) Representative western blot for subunits for oxidative phosphorylation complexes in skeletal muscle tissue. GAPDH is used as a loading control. Full-length membrane images can be found in Figure S6c-e. d) Quantification of skeletal muscle western blots in (c) for subunits for oxidative phosphorylation complexes normalized to GAPDH. Error bars SEM. One dot equals an individual animal.

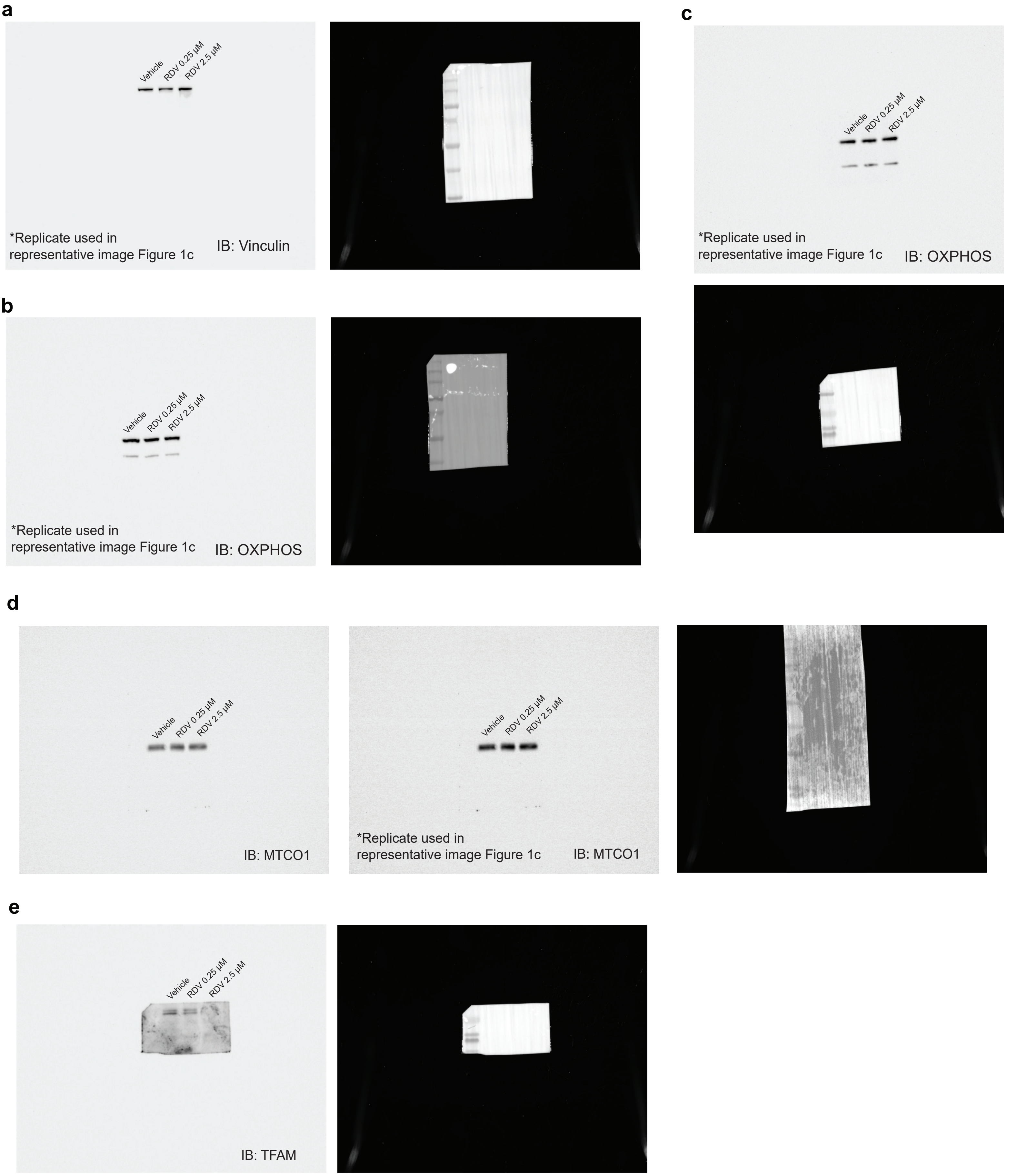

Supplementary Figure 2: Biological replicate 1 of Figure 1c, 1d.
(a-d) Full-length membrane images with ladder images of Figure 1c: oxidative phosphorylation expression level in Mv1Lu cells. (e) Full-length membrane images with ladder images of replicate 1 in Figure 1d: Mitochondrial transcription factor A expression level in Mv1Lu cells.

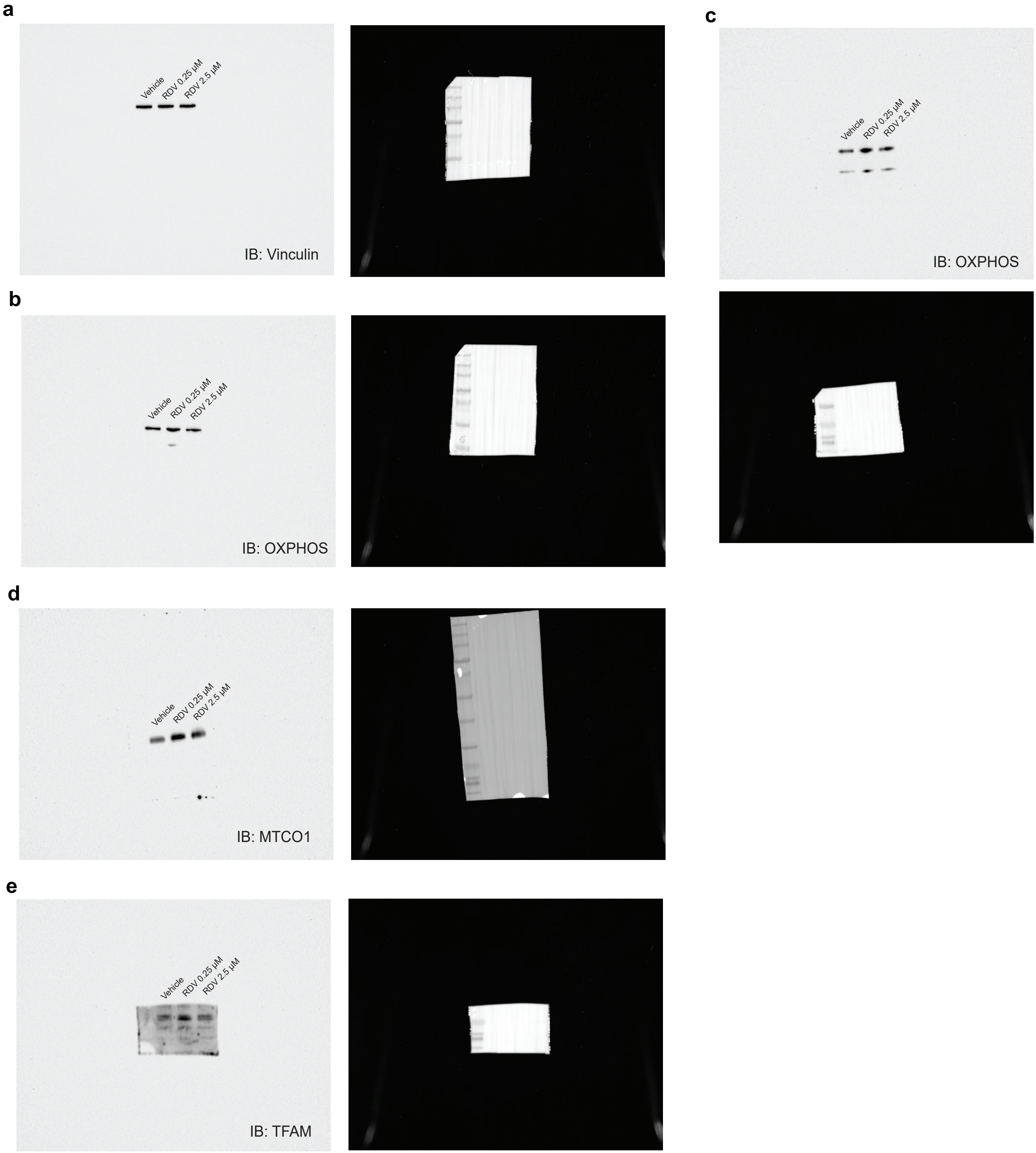

Supplementary Figure 3: Biological replicate 2 of Figure 1c,1d.
(a-d) Full-length membrane images with ladder images of replicate 2 in Figure 1c: oxidative phosphorylation expression level in Mv1Lu cells. (e) Full-length membrane images with ladder images of replicate 2 in Figure 1d: Mitochondrial transcription factor A expression level in Mv1Lu cells.

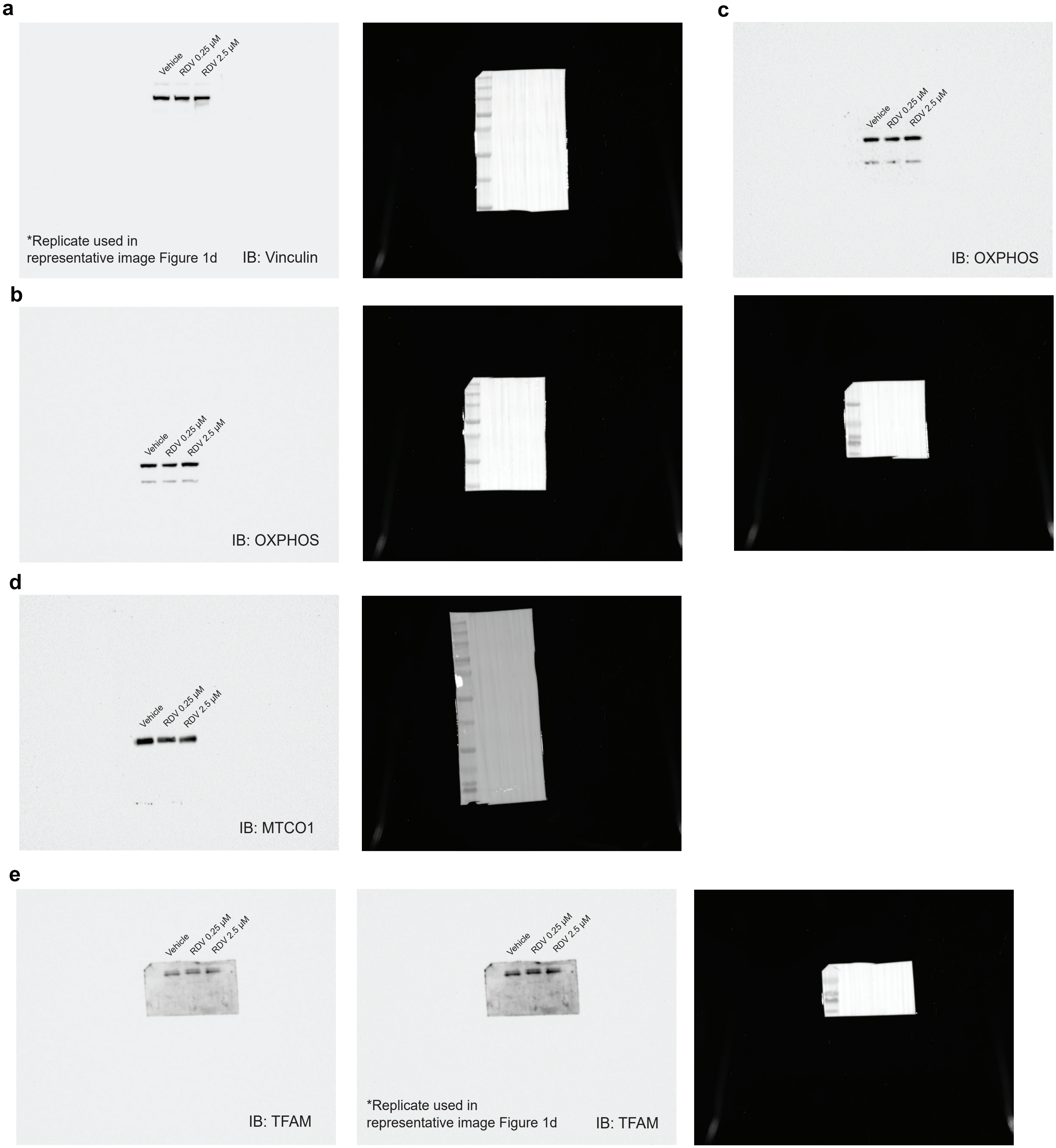

Supplementary Figure 4: Biological replicate 3 of Figure 1c,1d.
(a-d) Full-length membrane images with ladder images of replicate 3 in Figure 1c: oxidative phosphorylation expression level in Mv1Lu cells. (e) Full-length membrane images with ladder images of Figure 1d: Mitochondrial transcription factor A expression level in Mv1Lu cells.

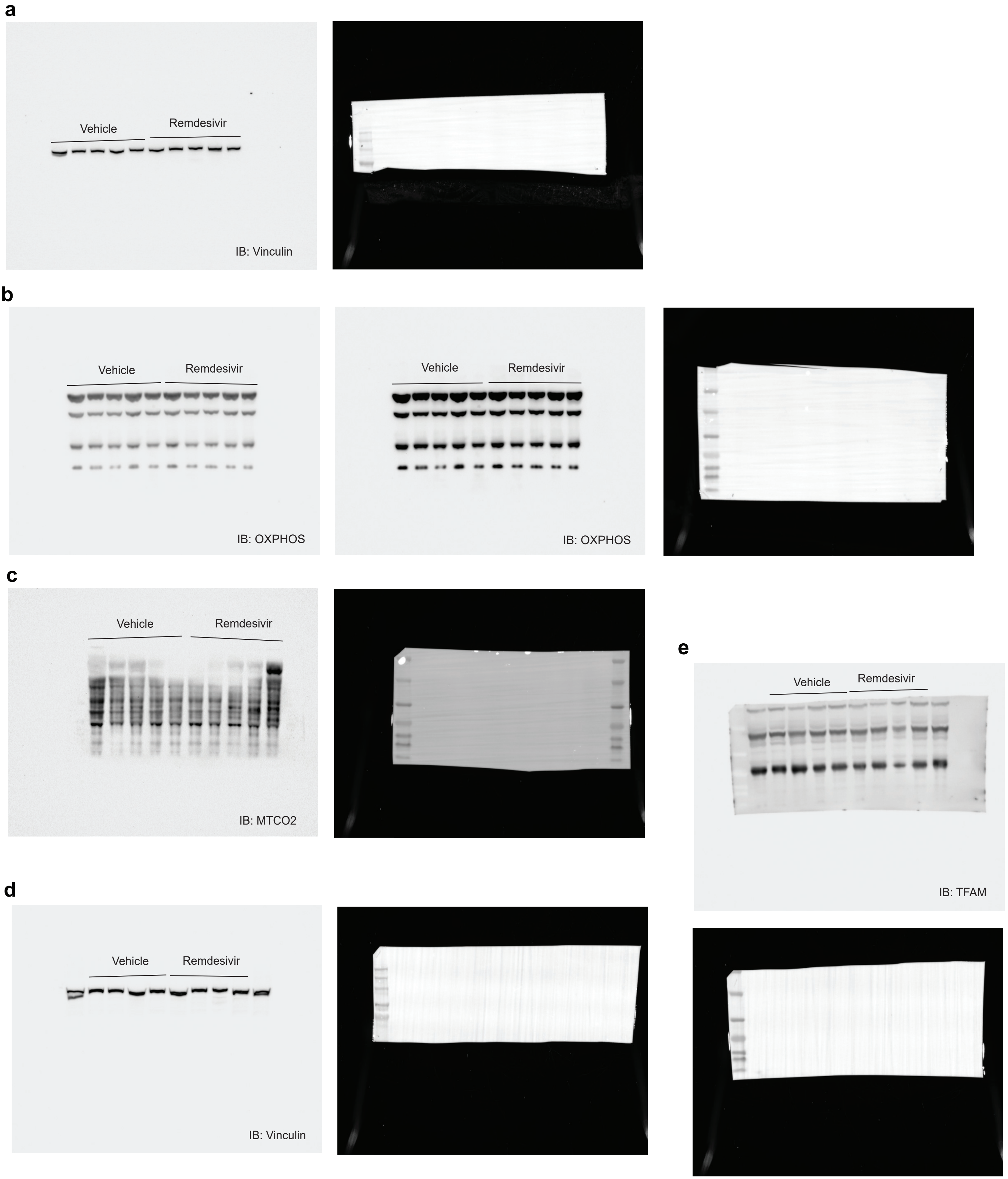

Supplementary Figure 5:
(a-c) Full-length membrane images with ladder images of Figure 3a: oxidative phosphorylation expression level in liver tissue. (d,e) Full-length membrane images with ladder images of Figure 3c: Mitochondrial transcription factor A expression level in liver tissue.

a

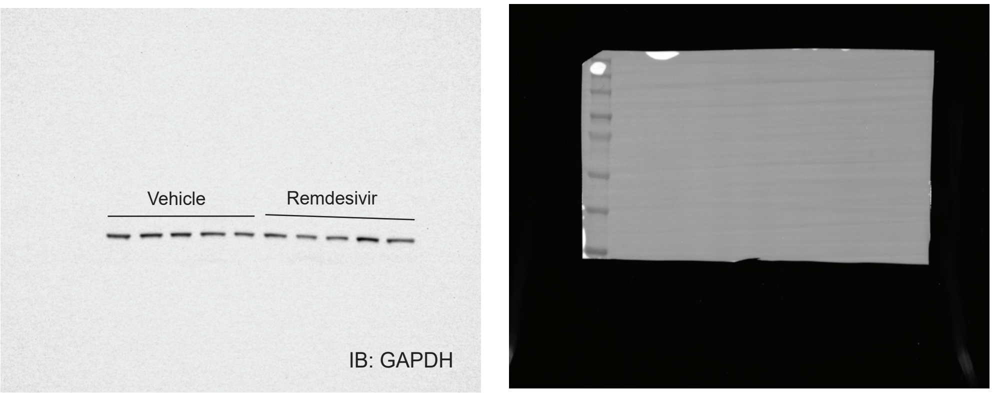

b

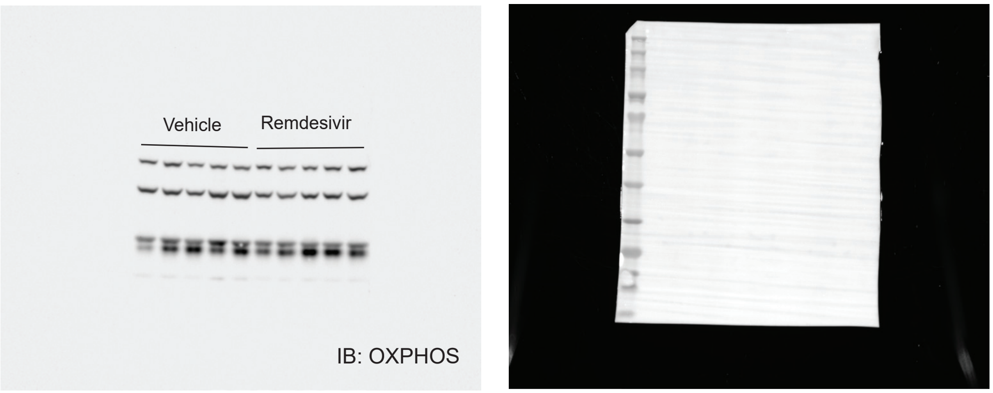

c

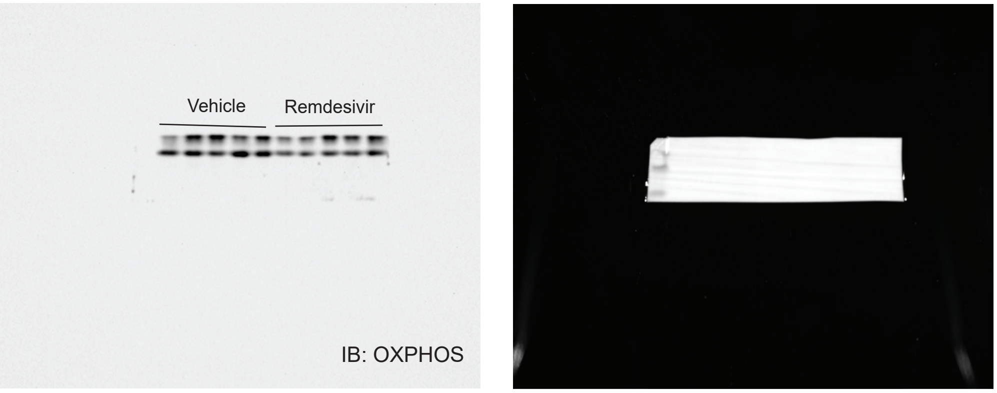

d

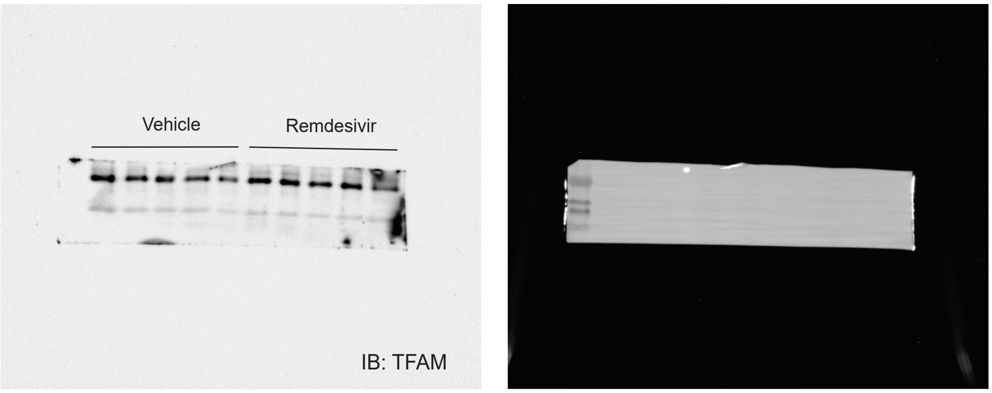

Supplementary Figure 6:
(a-d) Full-length membrane images with ladder images of Figure 5a: oxidative phosphorylation and mitochondrial transcription A expression level in lung tissue.

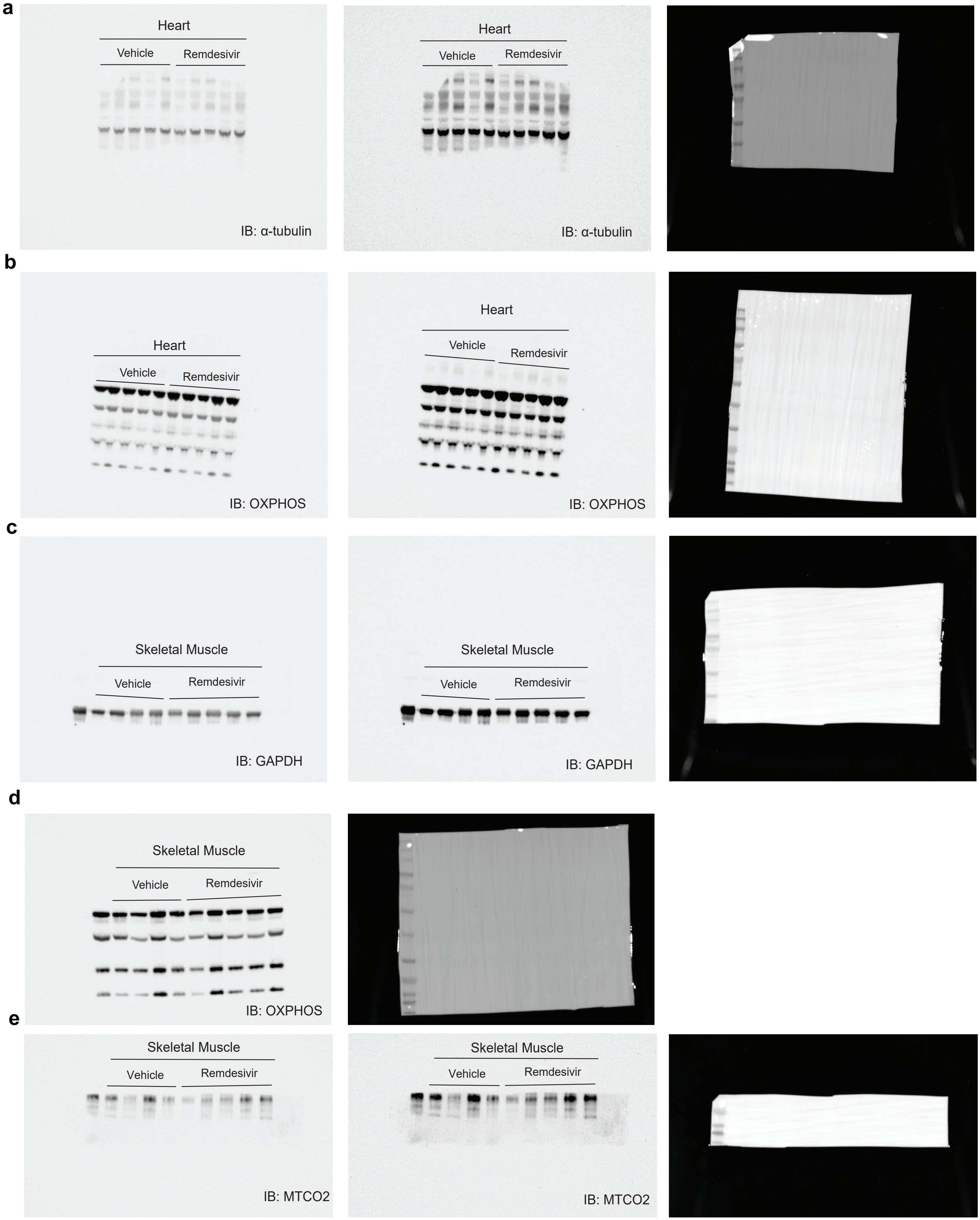

Supplementary Figure 7:

(a,b) Full-length membrane images with ladder images of Supplementary Figure 1a: oxidative phosphorylation expression level in heart tissue. (c-e) Full-length membrane images with ladder images of Supplementary Figure 1c: oxidative phosphorylation expression level in skeletal muscle tissue.
